# Identifying Datasets for Cross-Study Analysis in dbGaP using PhenX

**DOI:** 10.1038/s41597-022-01660-4

**Published:** 2022-09-01

**Authors:** Huaqin Pan, Vesselina Bakalov, Lisa Cox, Michelle L. Engle, Stephen W. Erickson, Michael Feolo, Yuelong Guo, Wayne Huggins, Stephen Hwang, Masato Kimura, Michelle Krzyzanowski, Josh Levy, Michael Phillips, Ying Qin, David Williams, Erin M. Ramos, Carol M. Hamilton

**Affiliations:** 1 RTI International, Research Triangle Park, NC USA; 2 National Center for Biotechnology Information, National Library of Medicine, National Institutes of Health, Bethesda, MD USA; 3 GeneCentric Therapeutics Inc., Durham, NC USA; 4 Levy Informatics, Chapel Hill, NC USA; 5 National Human Genome Research Institute, National Institutes of Health, Bethesda, MD USA

**Keywords:** Genetics research, Epidemiology

## Abstract

Identifying relevant studies and harmonizing datasets are major hurdles for data reuse. Common Data Elements (CDEs) can help identify comparable study datasets and reduce the burden of retrospective data harmonization, but they have not been required, historically. The collaborative team at PhenX and dbGaP developed an approach to use PhenX variables as a set of CDEs to link phenotypic data and identify comparable studies in dbGaP. Variables were identified as either comparable or related, based on the data collection mode used to harmonize data across mapped datasets. We further added a CDE data field in the dbGaP data submission packet to indicate use of PhenX and annotate linkages in the future. Some 13,653 dbGaP variables from 521 studies were linked through PhenX variable mapping. These variable linkages have been made accessible for browsing and searching in the repository through dbGaP CDE-faceted search filter and the PhenX variable search tool. New features in dbGaP and PhenX enable investigators to identify variable linkages among dbGaP studies and reveal opportunities for cross-study analysis.

## Introduction

Secondary analysis using multiple study datasets can validate findings from individual studies and generate more power to detect subtle and complex associations not possible by individual studies^1–7^. However, identifying the relevant datasets to include in a secondary analysis from publicly available data repositories can be challenging. One fundamental challenge in searching the metadata of archived biological datasets lies in the unstructured nature of the variable description. Additionally, variation in the semantic terms poses a barrier for data findability and reuse. For example, “drink regularly past month” and “alcohol consumption frequency last 30 days” can be recognized as comparable concepts by manual curation, but not by simple keyword search. Ultimately, heterogeneity in data collection, and especially the lack of standard measurement protocols used to collect data, restricts the ability to combine or harmonize data from multiple studies over time and therefore limits the overall impact of individual studies. Ideally, each funded study would have further scientific impact after its initial analysis through secondary analyses and meta-analyses designed to answer research questions requiring large patient populations.

The National Institutes of Health (NIH) Strategic Plan for Data Science (SPDS) promotes FAIR principles (i.e., **F**indable, **A**ccessible, **I**nteroperable, **R**eusable) to facilitate data sharing. Objective 2–3 of the SPDS, “Leverage Ongoing Initiatives to Better Integrate Clinical and Observational Data into Biomedical Data Science,” states that the NIH will “promote use of the NIH Common Data Elements Repository^8^”. Several NIH Common Data Element (CDE) resources have been established to address the data silo challenge^9–12^. PhenX (consensus measures for Phenotypes and eXposures), as one of the NIH CDE Repository projects, is a community-driven project to establish and promote the use of standard data collection protocols to improve the quality and consistency of data collection and to facilitate data sharing. The PhenX Toolkit shares measurement protocols that are recommended by experts. A subset of PhenX protocols is included in the NIH CDE Repository. Each standard protocol can itself be a CDE, and the protocols often contain a set of variables, which can also be used as CDEs to harmonize data. Incorporating standard measurement protocols at the study design stage can reduce the need for data harmonization over time by addressing the challenge of data harmonization at its source^13–17^. Retrospective data harmonization has many challenges and entails a labor-intensive process to combine data collected using different data collection protocols^18–24^. Use of CDEs can help identify comparable study datasets from multiple data sources and repositories, enable cross-study analysis, and reduce the burden of retrospective data harmonization^25,26^.

The PhenX Toolkit (https://www.phenxtoolkit.org) is a catalog of measurement protocols recommended for use by working groups of domain experts. In general, PhenX protocols are well-established, low-burden methods of collecting important data to assess phenotype and exposure data in studies involving human participants, including clinical, translational, genomic, and epidemiological studies. The protocols included in the PhenX Toolkit are relevant to a wide range of research domains^27–31^. Specialty collections in the PhenX Toolkit provide additional depth in specific research areas, such as Social Determinants of Health and COVID-19^32–36^. The database of Genotypes and Phenotypes (dbGaP) at the National Library of Medicine’s National Center for Biotechnology Information (https://www.ncbi.nlm.nih.gov/gap/) was created to provide a common location for storage of data from NIH-funded genomics-based and other studies. As a controlled access data repository that archives and distributes data from research studies investigating the interrelated nature of genotypes, phenotypes, and exposures^37,38^ dbGaP has adapted to manage a wide and continually expanding variety of data types since its creation. It supports the NIH genomic data sharing policy^39^ by allowing for the secondary use of data by investigators who have an approved research proposal consistent with the data use limitations for each dataset. However, most studies present in the dbGaP database were completed prior to the introduction and recommendation of CDEs, and before the first release of the PhenX Toolkit in 2009. The lack of CDEs and the heterogeneity of terminology among dbGaP studies increases the monetary and personnel burdens for investigators, who must manually review hundreds of data elements to identify studies which may be suitable for data harmonization and meta-analysis.

In this paper, we describe the process for mapping PhenX CDEs consisting of either protocols or individual variables to dbGaP study variables; the development of tools to help investigators find datasets using these linkages; and new options available for the identification of CDEs when submitting studies to dbGaP. PhenX protocols (e.g., “Tobacco - Age of Initiation of Use - Adolescent” [PX030701]) consist of a list of variables to collect data about phenotypes and exposures (e.g., PX030701_First_Cigarette_Smoking_Age). The dbGaP study datasets (e.g., Jackson Heart Study [JHS] Cohort, phs000286) consist of variables recording phenotype data (e.g., TOBA2, phv00128497). The PhenX – dbGaP variable mapping process compares the PhenX variables with the dbGaP variables and identifies all the dbGaP variables that can be mapped to relevant PhenX variables. The addition of PhenX CDE linkages at the variable level enables investigators who visit dbGaP to identify linked variables across studies which were not retrievable using keyword search, thus enhancing the findability of linked datasets.

## Results

### Linking studies via PhenX-dbGaP variable mapping

In this paper, we report the mapping results from the February 28, 2017, data freeze, which includes the June 8, 2016, release of dbGaP and the August 30, 2016, release of the PhenX Toolkit. The results of this mapping include 13,653 dbGaP linked variables that come from 521 dbGaP studies using PhenX variables (see “Mapping PhenX variables to dbGaP study variables” in Methods for more detail). These variable mappings include “many-to-many” relationships because both PhenX measurement protocols and dbGaP studies have redundant variables (Table 2). For example, the PhenX variables collecting information on “History of Cancer” are present in “Personal History” for multiple disease and condition measurement protocols. Similarly, some dbGaP variables in longitudinal studies were measured at multiple visits.

### Identifying opportunities for cross-study analysis

#### Scenario 1

**Fig. 1:**
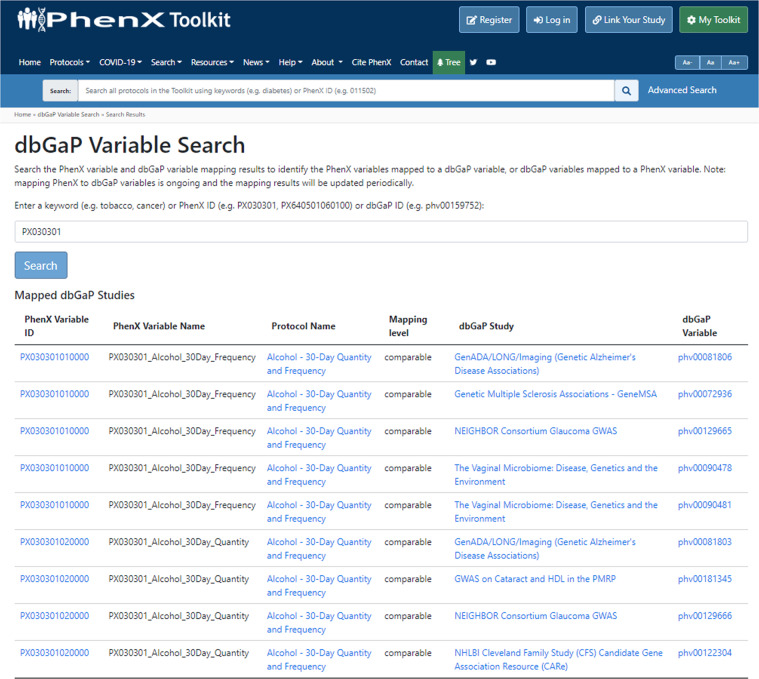
PhenX “dbGaP Variable Search” Tool. This tool (https://www.phenxtoolkit.org/vsearch) allows users to search keyword, PhenX identifiers, or dbGaP identifiers to find the PhenX-dbGaP variable mappings. For example, searching PhenX ID “PX030301” returns 9 dbGaP variables from 6 dbGaP studies with links to dbGaP.

**Fig. 2:**
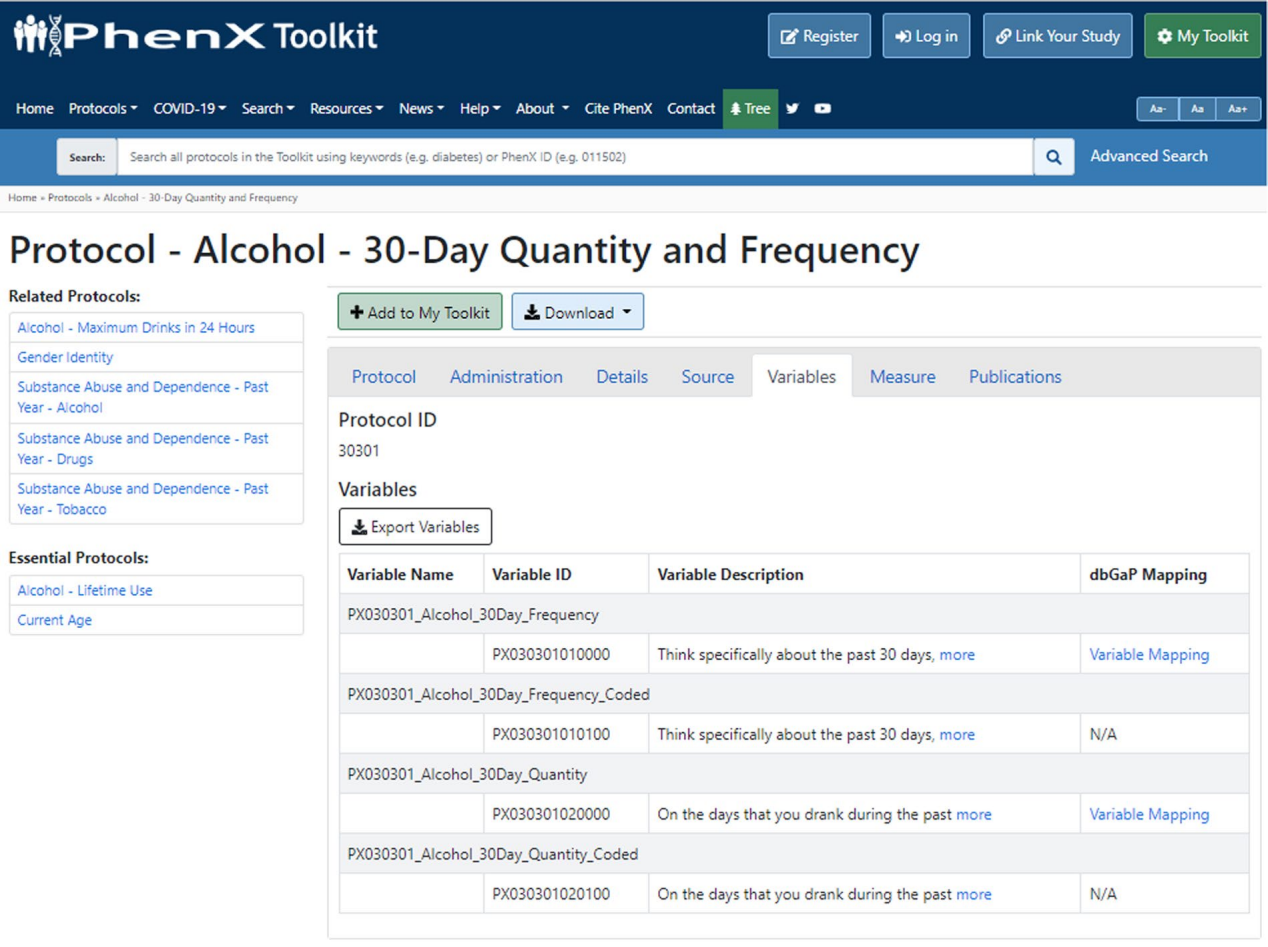
PhenX protocol page contains mappings to dbGaP variables. At the PhenX protocol page, Alcohol - 30-Day Quantity and Frequency” (https://www.phenxtoolkit.org/protocols/view/30301), navigating to the “Variable” tab shows available mappings to dbGaP variables listed for each PhenX variable in the measurement protocol.

PhenX users collecting alcohol use data identify dbGaP studies linked by variable mappings. First, the user can use the PhenX “dbGaP Variable Search” under “Search” (https://www.phenxtoolkit.org/vsearch) to search keywords or PhenX or dbGaP identifiers. For example, searching PhenX measurement protocol ID “PX030301” (which represents the protocol titled “Alcohol - 30-Day Quantity and Frequency”) returns nine dbGaP variables from six dbGaP studies with links to dbGaP web pages (Fig. 1). The PhenX variable ID links to the measurement protocol page providing complete variable information, and the dbGaP results link to the dbGaP study and variable pages, respectively. Additionally, users browsing the PhenX Toolkit can visit the individual PhenX measurement protocol page, for example, “Alcohol - 30-Day Quantity and Frequency” (https://www.phenxtoolkit.org/protocols/view/30301). Navigating to the “Variable” tab shows available mappings to dbGaP variables listed for each PhenX variable in the measurement protocol (Fig. 2).

#### Scenario 2

**Fig. 3:**
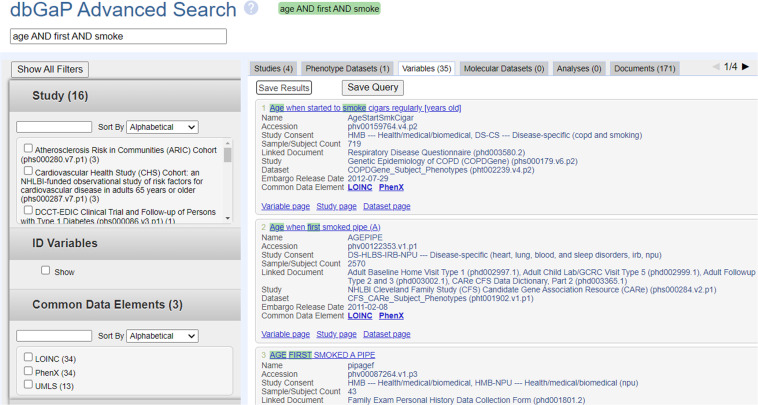
Find PhenX mappings in the dbGaP SOLR-faceted “Advanced Search” tool. dbGaP variables with PhenX mappings can be found in the SOLR-faceted “Advanced Search” tool, https://www.ncbi.nlm.nih.gov/gap/advanced_search/. Searching “age AND first AND smoke” returns 35 variables in the “Variables” tab where PhenX is listed under the “Common Data Elements” facet on the left.

A dbGaP user is interested in all study data sets collecting “Age when first smoke cigarettes.” Using the dbGaP SOLR-faceted “Advanced Search” tool (https://www.ncbi.nlm.nih.gov/gap/advanced_search/), users can enter search keywords “age” AND “first” AND “smoke” into the search field. This returns 35 variables in four studies annotated with the “Common Data Elements” (Fig. 3). Clicking the “Variables” tab (to the right of the default “Studies” tab), reveals four pages of results, which the user can navigate between using the black arrows at the top of the search results page. This includes the variable TOBA2 on page 3 (https://www.ncbi.nlm.nih.gov/projects/gap/cgi-bin/variable.cgi?study_id=phs000286.v6.p2&phv=128497).

#### Scenario 3

A user annotates CDE source during dbGaP data submission. In addition to the retrospective mapping of PhenX CDEs to dbGaP studies already included in the database, we modified the dbGaP study data submission process to capture PhenX CDE use for new studies being submitted to dbGaP. Two additional columns were added to the dbGaP data dictionary to record CDE linkage: VARIABLE_SOURCE and SOURCE_VARIABLE_ID. Using these columns, a researcher depositing data in dbGaP can indicate that a submitted variable used a specific PhenX protocol (e.g., VARIABLE_SOURCE = “PhenX” and SOURCE_VARIABLE_ID = “PX130301”). These columns are intended to capture mappings that are tagged at the “identical” mapping level, which is reserved specifically for the prospective use of PhenX measurement protocols in data collection. The other two mapping levels (“comparable” and “related”) are for retrospective mapping of variables by the PhenX curation teams (Table 1).

**Table 1:** Definitions of mapping levels.

**Identical**	Variables that are immediately ready for direct harmonization between datasets, without any transformation needed to combine the data. At the time of curation, this term was reserved for prospective, investigator self-identified use for future submissions to the dbGaP database.
**Comparable**	Two variables that were conceptually similar and that contain data that can be directly harmonized or compared after a simple logical or mathematical transformation.
**Related**	Bioassay variables and other instances when the methods for data collection may be distinct. This distinction is to alert investigators that they should review the methods carefully before proceeding with transformation of the data for harmonization.

## Discussion

Unique challenges exist in mapping PhenX variables to dbGaP study variables. The PhenX-dbGaP mapping compares the PhenX and dbGaP variable description fields, which are both stored as a string of unstructured terms, often covering multiple concepts. For example, the PhenX variable “In your entire life, have you had at least 1 drink of any kind of alcohol, not counting small tastes or sips?” (PX030101010000) is comparable to dbGaP variables such as “Has patient ever consumed at least one (alcoholic) drink/week for one year or more” (phv00034229) or “Was there ever a period when you drank alcoholic beverages regularly?” (phv00161469). A search using keywords representing a unique concept in the concise space of one or two words (e.g., diabetes or lung cancer) can yield good results, but using a lengthy string to impose specificity representing multiple concepts (alcohol, time frame, qualifiers) poses a challenge.

We tested available Natural Language Processing (NLP) tools during the mapping effort in 2016^40^ and found that a common problem in the predicted result sets was the inclusion of many false positive results due to the unstructured multi-concept nature of dbGaP variable description. We determined that weeding out the false positives would be managed most effectively by a manual curation process. One possible method of applying NLP more effectively to map these data would be to generate enough relevant training data to improve performance sufficiently to reduce the amount of effort needed for manual curation.

Because manual curation is not scalable and because both the PhenX and dbGaP databases continue to grow, development of NLP tools that can be applied to the variable mapping effort is a compelling approach to consider. Recently, there have been significant advances in the development and application of NLP algorithms^41–48^. The performance of the NLP tools relies on the size, quality, and content coverage of a well-annotated and structured training set. The set of mapping results presented here provides an invaluable training and test dataset for the future application of an NLP tool to refine algorithms for recognizing multi-concept strings.

Variable mapping as annotation of CDEs increases findability in data repositories. There are many unidentified opportunities for secondary analysis of studies measuring phenotypic aspects of complex diseases in publicly available data repositories. To take advantage of the large amount of data present and publicly available in repositories, NIH has been encouraging data reuse through multiple funding opportunities^49–52^. Previously, identifying datasets for inclusion in any secondary analysis, such as a meta-analysis, had been a laborious process that required hours of manual review to identify datasets with potential for harmonization due to heterogeneity of variable semantics and differences in data collection methods. The results reported here use PhenX variables as a set of CDEs to annotate data elements in dbGaP studies and, furthermore, provide an additional way of indexing dbGaP studies for more effective browsing and searching. Increasing the annotation of CDE data elements within the dbGaP repository will streamline the process of study identification and data harmonization. The linkage and annotation, together with the submission and search tools developed, addresses this barrier of missing linkage due to semantic variation and increases the searchability of CDEs in the dbGaP repository.

The dbGaP search at the variable level often returns a high proportion of false positive results (e.g., in matching a single keyword without the context, the chemical “lead” can’t be distinguished from the “lead” in electrocardiograph) and/or a high proportion of false negative results by missing relevant results using alternative semantics (e.g., smoking vs cigarette). The newly developed CDE browsing facets (dbGaP) and search tool helps the user to retrieve studies with more specificity and sensitivity and decreases investigator burden by reducing the time required for manual review. dbGaP users can now identify relevant study datasets in dbGaP, which was not possible before. Additionally, when PhenX users browse the PhenX measurement protocols to decide whether to include them during study design, the availability of linked dbGaP studies enables them to identify publicly available datasets for future cross-study analysis. With insightful CDE inclusion during study design and CDE annotation in data repositories, individual studies can broaden their impact with lower costs through reuse of other study datasets in data repositories.

Rich metadata enhances interoperability. The FAIR principles advocate for standard metadata practices, such as including qualified references to other metadata, to increase interoperability^53^. The PhenX Toolkit is designed to improve consistency of data collections and, thus, supports data interoperability and reusability^53,54^. To date, the adoption of PhenX measurement protocols in prospective studies has been recommended in 523 NIH Funding Opportunity Announcements and 28 Notices. Using PhenX variables as CDEs to link retrospective studies in dbGaP, as reported in this paper, provides another important resource to identify studies with sufficient commonality to support cross-study analysis. Moving forward, to maximize the value of common measures used in various studies and to enhance interoperability, PhenX plans to expand this effort of annotating CDEs among studies to additional NIH Data Repositories and Data Commons that are willing to collaborate. Additionally, we propose to utilize Logical Observation Identifiers Names and Codes (LOINC) standards and adopt the FHIR (Fast Healthcare Interoperability Resources) specification to enhance PhenX measurement protocols’ interoperability for integration into the NIH data ecosystem.

## Methods

### Mapping PhenX variables to dbGaP study variables

To help streamline the mapping process, we developed an R Shiny search tool to help organize and refine the dbGaP variable dataset for curation. The tool allowed the PhenX curation team to use Boolean logic (AND, OR, NOT) to combine search queries to scan variable descriptions for multiple concepts, and multiple Boolean statements could be combined to create more complex queries. This team of curators manually evaluated these initial keyword search results to decide whether each suggested pair of the PhenX-dbGaP variables were conceptually equivalent to qualify as “comparable” or “related” as a mapping. The determination was based upon a set of criteria measuring the potential for harmonization of the datasets collected, as described below and summarized in Table 1. Each set of potentially mapped PhenX-dbGaP variables was then reviewed by independent curators to ensure consistent and high-quality mapping. Any discrepancy was resolved in a group discussion. A “comparable” mapping level was defined as two variables that were conceptually similar and that contained data that can be directly harmonized or compared after a simple logical or mathematical transformation, including a categorical approximation or grouping, as listed in Table 2. A “related” mapping level was assigned to bioassay variables and other instances when the methods for data collection may be distinct. This distinction is to alert investigators that they should review the methods carefully before proceeding with transformation of the data for harmonization. For example, “Concentration of Immunoglobulin E” and “Immunoglobulin E (EDTA plasma)” bioassays often measure the same analyte, but the methodology may differ despite the dataset’s appearing directly harmonizable in terms of measurements and units.

**Table 2:** Examples of “comparable” mapping level with various scope and diversity.

**Variables with different units that require a simple algorithmic transformation to convert**	
Average number of packs smoked per day	How many cigarettes per day do/did you smoke?
Free-form numeric annual income number	Quantitative income category of “$35,000–$50,000” or “below poverty line”
**Variables addressing similar concepts with different semantic terminology**	
Have you had any of these clinician-diagnosed illnesses—Stroke?	Stroke ever diagnosed
	Subject’s history of disease—stroke
	Hemorrhagic stroke
	Ischemic stroke
	CVA (Cerebrovascular accident)
	Cerebral stroke
	Cerebrovascular accident
	Cerebrovascular apoplexy
	Cerebrovascular stroke
	Brain Vascular Accident

### New dbGaP variable search tool released in PhenX Toolkit

To make PhenX-dbGaP mapping accessible, we developed a dbGaP Variable Search Tool at the PhenX Toolkit website (https://www.phenxtoolkit.org/vsearch). We loaded the mapping results into the PhenX Toolkit database and used PHP and JavaScript to enable the web interface. To find PhenX-dbGaP variable mappings, searches can be run using keywords, PhenX measurement protocol ID, PhenX variable ID, or dbGaP variable ID.

### New search tools and features released in dbGaP

PhenX-dbGaP variable mappings are loaded by dbGaP and accessed through a SOLR-based faceted search (Fig. 3.) To facilitate searching of PhenX-dbGaP variable linkages, we additionally indexed PhenX variable ID, name, and descriptions for dbGaP studies and variables. With this development, searches can be executed based on PhenX properties (e.g., PhenX variable name, accession, keywords) or dbGaP properties (e.g., dbGaP variable name, accession, description). Although we implemented this strategy for PhenX variables specifically, we adopted it more generally to embrace CDE linkages from multiple initiatives such as Logical Observation Identifiers Names and Codes (LOINC) and Unified Medical Language System (UMLS). The CDE mappings that enable searching are also represented as links displayed on each dbGaP variable page. These CDE links can be authored by either the data submitter via a data dictionary or another party doing a retrospective mapping (e.g., the PhenX team).

In addition to facilitating direct CDE (e.g., PhenX) to dbGaP variable mapping, we modified dbGaP’s study data submission process to include two additional columns to dbGaP’s data dictionary: VARIABLE_SOURCE and SOURCE_VARIABLE_ID. Using these columns, a researcher depositing data in dbGaP can indicate that a submitted variable is mapped to a specific PhenX measurement protocol, such as VARIABLE_SOURCE = “PhenX” and SOURCE_VARIABLE_ID = “PX130301.” These columns are intended to capture mappings that are tagged at the “identical” mapping level, which is reserved specifically for the prospective, submitter-identified use of PhenX measurement protocols in data collection. The other two mapping levels (comparable and related) are reserved for retrospective mapping of variables.

In summary, the use of CDEs can help identify comparable study datasets from multiple data sources and repositories, enable cross-study analysis, and reduce the burden of retrospective data harmonization. We presented an approach of using PhenX measurement protocols and variables to identify CDEs linking across the study-specific variables in dbGaP studies. Both dbGaP and PhenX have developed browse and search tools to access variables and studies linked by the mappings. Users of dbGaP and PhenX can browse and search variables of interest to find other studies that have collected data using the same variable. This development adds features in dbGaP and PhenX that help investigators identify opportunities for cross-study analysis and maximize research benefits beyond the original objective of a single study.

## Data Availability

Data sharing is not applicable to this article as no datasets were generated or analyzed during the current study.

## Code availability

Code sharing is not applicable to this article as the development distributed linked metadata (dbGaP variable ID linked to PhenX variable ID) in existing databases and features from PhenX and dbGaP.
